# Volumetric‐modulated arc therapy and intensity‐modulated radiation therapy treatment planning for prostate cancer with flattened beam and flattening filter free linear accelerators

**DOI:** 10.1002/acm2.12168

**Published:** 2017-08-30

**Authors:** Marius Treutwein, Matthias Hipp, Oliver Koelbl, Barbara Dobler

**Affiliations:** ^1^ Department for Radiotherapy Regensburg University Medical Center Regensburg Germany; ^2^ Klinikum St. Marien, Strahlentherapie Amberg Germany

**Keywords:** flattening filter free, IMRT, prostate cancer, VMAT

## Abstract

This study on patients with localized prostate cancer was set up to investigate valuable differences using flattened beam (FB) and flattening filter free (FFF) mode in the application of intensity‐modulated radiotherapy (IMRT) and volumetric‐modulated arc therapy (VMAT). For ten patients, four different plans were calculated with Oncentra planning system of Elekta, using Synergy machines: IMRT and VMAT, with and without flattening filter. Homogeneity and conformity indexes, dose to the organs at risk, and measurements of peripheral dose and dosimetric plan verification including record of the delivery times were analyzed and statistically evaluated. The indexes for homogeneity and conformity (CTV and PTV) are either advantageous or not significantly different for FFF compared to FB with one minor exception. Regarding the doses to the organs at risk and the measured peripheral dose, equivalent or lower doses were delivered for FFF than with FB. Furthermore, the delivery times were significantly shorter for FFF. VMAT compared to IMRT reveals benefits or at least equivalent values. VMAT‐FFF combines the most advantageous plan quality parameters with the shortest delivery times and reduced peripheral dose and is therefore recommended for the given equipment and cancer localization.

## 1 INTRODUCTION

Fluence modulating treatment techniques like intensity‐modulated radiotherapy (IMRT) and volumetric‐modulated arc therapy (VMAT) are well established for many tumor sites. These techniques now enable the clinical application of linear accelerators (linacs) with flattening filter free mode (FFF), thus obtaining a much higher dose rate than in the standard flattened beam mode (FB). The first reported clinical investigations of FFF referred to radiosurgery^1^ and aimed for shorter treatment times. A reduction of the treatment time reduces the probability of intrafraction motion of the target and organs at risk, which has been demonstrated to be not negligible for the treatment of prostate cancer.^2^, ^3^, ^4^, ^5^, ^6^, ^7^, ^8^ Another property of FFF is the diminution of head scatter, as the flattening filter is one of its main sources.^9^, ^10^ This should affect the peripheral dose (PD), which is a factor of secondary cancer probability. Effects of FFF on treatment plans with IMRT and VMAT technique are compared to FB plans in this planning study, evaluating treatment times, plan quality, and PD.

Although there are some other publications regarding the treatment of patients with prostate carcinoma using linacs with FFF mode, they refer to other types of rotational technique and manufacturers and different treatment planning systems (TPS).^11^, ^12^, ^13^ However, the design of the linac head affects the PD^14^ and the penumbra of the beam. The desktop software, the hardware of the linac, and the TPS influence the treatment time. Kry et al.^15^ showed that there is also a dependency of the PD on the field size and the amount of modulation. Therefore investigations with the same or similar equipment,^16^, ^17^ but different targets cannot simply be transferred. This work demonstrates for the first time a comparison of FB and FFF plans for prostate cancer therapy with the given equipment of linacs and TPS.

## 2 MATERIAL AND METHODS

### 2.A Patients, regions of interest (ROI), and dose prescription

Ten consecutive patients with histologically proven, previously untreated localized prostate cancer were included in this planning study. All patients had given their informed consent to take part in the study. DICOM data sets including delineated regions of interest were taken from a former investigation.^18^ The mean age of the patients was 71 yr. For the planning CT study, they were immobilized in supine position in a vacuum mattress (Blue‐BAG™ BodyFIX^®^, Medical Intelligence, Schwabmünchen, Germany) according to Boehmer et al.^19^ In each slice with an effective distance of 2.5 mm, the following volumes of interest were delineated following the description of Bos et al.^20^: As target volumes the gross target volume (GTV: prostate gland and seminal vesicles), the clinical target volume (CTV: 5‐mm three‐dimensional margin added to the GTV excluding the rectal volume), the planning target volume (PTV: 10‐mm three – dimensional margin added to the GTV without respect to the rectum), and as organs at risk (OAR) the rectal volume (according to Guckenberger et al.^21^), urinary bladder and femoral heads. To achieve high plan quality, two additional volumes were constructed. First, the PTV with an added margin of 5 mm was subtracted from the rectum volume, resulting in the posterior part of the rectum; second, the CTV was subtracted from the PTV (PTV − CTV) to model the dose gradient in this region.

Criteria were formulated to build a complete set of accepted values which represents the dose prescription in the sense of ICRU report 83:^22^ The average dose to the CTV should be in the range of 71.5 Gy to 73.7 Gy, allowing a deviation of 1.5% in the total dose. For the PTV, 56.4 Gy were set as acceptance value for D_98%_^PTV^. For the OAR, we required the volume of the rectum receiving up to 70 Gy to be smaller than 20%, the volume of the urinary bladder and the femoral heads receiving up to 50 Gy to be smaller than 50%.

### 2.B Linear accelerator

The linac used in the TPS and for the measurements is an Elekta Synergy™ with Agility™ head (Elekta AB, Stockholm, Sweden), which is equipped with 80 interdigitating leaf pairs, having 5 mm leaf width projected to the isocenter. 6 MV photons were applied in FB and FFF mode. It has been shown earlier that the beam quality is very similar for both modes,^23^ as it is common for Elekta linacs.^24^ The maximum dose rate is 500 monitor units (MU) per minute in FB mode and 1700 MU per minute in FFF. The desktop software is Integrity 3.1 and the record and verify system Mosaiq 2.50.

### 2.C Treatment planning system

The treatment planning was performed with Oncentra^®^ External Beam v4.5 (Nucletron^®^, an Elekta AB). The system has been commissioned especially for VMAT application with BeamModulator™ head first^25^ and later also for Agility™ head.^23^ The software supports variable gantry speed, which was set to a maximum value of 6.0 degree per second, and variable dose rate with a set minimum value of 20 MU per minute. Static and dynamic minimum leaf gaps were set to 1.00 cm. Calculations were performed using the collapsed cone algorithm. The optimizer module in Oncentra^®^ for IMRT and VMAT has been developed by RaySearch Laboratories (Stockholm, Sweden), as the similar SmartArc module integrated in the Pinnacle³ TPS (Philips, Amsterdam, Netherlands), and also the RayArc module in the proprietary RayStation TPS. For IMRT optimization, the direct step‐and‐shoot algorithm was used.^26^

### 2.D Planning

The dose optimization parameters and fractionation schedule were taken from the above mentioned study:^18^ The plans were set up with simultaneous integrated boost in 33 fractions aiming for 59.4 Gy minimum dose to the PTV (being 105% of the acceptance value for D_98%_^PTV^ of 56.4 Gy as described above) and 71.0 Gy minimum dose and 74.2 Gy maximum dose to the CTV, which was used as the boost treatment volume. The average value of minimum and maximum dose is 72.6 Gy. This corresponds to a single fraction dose of 2.2 Gy. The CTV objectives were set stronger than clinically achievable to force the DVH to a steep downfall and to get similar average dose values for each single plan. Setting a specific dose value to a dose point or average dose of a volume is not possible in the optimizer module of the Oncentra^®^ TPS. The same set of dose volume objectives (DVO) has been used for both modes, FB and FFF, IMRT as well as VMAT to make the results comparable^18^, ^27^, ^28^ (Table 1). For the same reason, individual plan optimizations with variations of weights or objectives have not been performed. The surrounding dose fall‐off objective is used to model the dose gradient from the surface of the PTV into the normal tissue.^29^ For all plans, the resulting average dose in the CTV was set to 100%. No rescaling of this dose to a specific dose value has been performed, as this may result in additional dose to the normal tissue and OARs which are part of the optimization process as well.^30^ As also stated for another TPS,^31^ the dose to the target is a free parameter of the cost function. Therefore, the resulting average dose in the CTV was taken as specification dose, thus representing the direct result of the objective function.

**Table 1:** Dose volume objectives used in the TPS Oncentra

ROI	Type	Dose level (Gy)	Volume (%)	Weight
CTV	Minimum dose	71.0	100	3000
CTV	Maximum dose	74.2	0	3000
PTV–CTV	Minimum dose	59.4	100	3000
PTV–CTV	Maximum dose	71.0	0	3000
Urinary bladder	Maximum dose volume	50	50	1000
Rectum	Maximum dose volume	70	20	1000
Rectum	Maximum dose volume	50	60	1000
Rectum	Maximum dose	74.2	0	1000
Posterior rectum	Maximum dose	50	0	1000
Left and right femoral head	Maximum dose volume	50	50	300
Outline	Surrounding dose fall‐off	59.4 to 29.7 within 5 mm	–	500

The center of the CTV was positioned to the isocenter. The calculation grid had a resolution of 0.25 cm.

The IMRT planning followed^26^ using seven equispaced beams with gantry angles of 0°, 51°, 103°, 154°, 206°, 257°, and 309°. The collimator was set to 0°. Additional parameters were adjusted: At least six open leaf pairs, a maximum number of 60 segments for the complete plan, a minimum number of 4 MU per segment, and the minimum field size was set to 9.0 cm².

The VMAT optimization used parameters which were deduced from our above mentioned earlier investigation:^18^ gantry single arc rotation from 182° to 178° with gantry spacing of 4° between two control points, collimator angle 45°, and a maximum delivery time of 110 s.

The planning was performed for the complete study by the same specialist medical physicist. After the first optimization and final dose calculation, a second cycle of optimization and dose calculation followed for each plan.

### 2.E Plan evaluation and statistics

For the evaluation of the plan quality, the following parameters were regarded. The homogeneity index HI in the CTV was defined according ICRU report 83^22^ HI: = (D_2%_^CTV^–D_98%_^CTV^)/D_av_^CTV^. D_2%_^CTV^ and D_98%_^CTV^ are the dose to 2% and 98% of the CTV, respectively. D_av_^CTV^ is the average dose to the CTV, which was set to 100%.

The conformity indexes in the CTV (CI^CTV^) and in the PTV (CI^PTV^) were calculated according to Paddick.^32^ CI^CTV^: = TV_71 Gy_^2^/(V_71 Gy_ × V^CTV^) and CI^PTV^: = TV_59.4 Gy_^2^/(V_59.4 Gy_ × V^PTV^) use the minimum values of Table 1 prescribed to the CTV (71 Gy) and PTV (59.4 Gy), respectively. TV_71 Gy_ and TV_59.4 Gy_ describe the volumes within the corresponding target receiving these dose values, and V_71 Gy_ and V_59.4 Gy_ are the equivalent volumes within the patient contour. V^CTV^ and V^PTV^ are the target volumes. D_98%_^PTV–CTV^ — the dose to 98% of the PTV − CTV — was regarded as parameter for the minimum dose to the PTV.

The following dose and dose volume values were evaluated: The dose exceeded by 50% of the volume to the urinary bladder D_50%_^UB^, to both femoral heads (D_50%_^lFH^ and D_50%_^rFH^) and to the rectum volume D_50%_^R^ and the dose value to the posterior part of the rectum D_2%_^Rpost^ which is exceeded by 2% of the volume. Additional parameters were taken from the QUANTEC review summary:^33^ The percentage of volume receiving less than a specified dose value, taking 70 Gy, 60 Gy, and 50 Gy for the rectum (V_70 Gy_^R^, V_60 Gy_^R^, V_50 Gy_^R^) and 70 Gy and 65 Gy for the urinary bladder (V_70 Gy_^UB^, V_65 Gy_^UB^). Furthermore, the total number of MU was recorded.

The statistical analysis was performed using the Wilcoxon test in IBM^®^ SPSS^®^ Statistics 23 (IBM Corporation, Armonk, NY, USA). The test was run with a significance level of 0.05. First, the FFF plans were compared to the FB plans for both IMRT and VMAT separately. Second, the IMRT and VMAT plans were compared, broken down into FFF and FB.

### 2.F Measurements

All plans were dosimetrically verified with a 2D‐array MatriXX Evolution (IBA, Schwarzenbruck, Germany) in stacks of solid water, type RW3 (PTW, Freiburg, Germany) using a hybrid technique as described previously^18^, ^34^, ^35^. The array was positioned in the horizontal isocenter plane and connected to a gantry angle sensor attached to the gantry. Every 200 ms, the gantry angle and a dose matrix were acquired by the software OmniPro I'mRT v. 1.7a of the same manufacturer automatically. A correction factor matrix was used to correct the matrices for angular dependencies, including couch attenuation. The sum of the corrected matrices was compared to the calculated dose by gamma evaluation^36^ with a dose tolerance of 3% of the maximum dose and a distance to agreement of 3 mm. The dose calculations in the TPS were performed with a dose grid of 1.5 mm in the measurement plane. We restricted the area of the evaluation to dose values above 10% of the maximum dose as recommended by Ezzell et al.^37^ The percentage of pixels in range (γ ≤ 1) of the gamma evaluation was registered.

**Figure 1:**
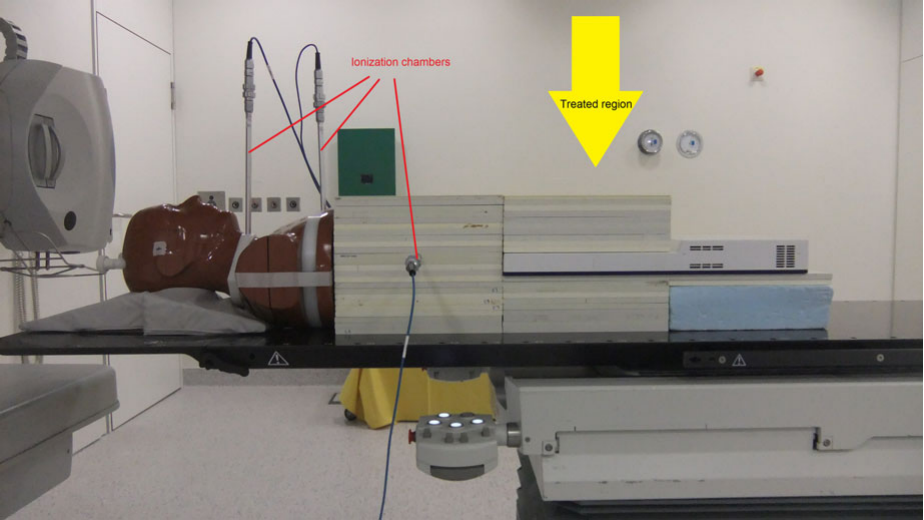
Measurement setup with 2D‐array, ionization chambers and Alderson phantom.

For the evaluation of the PD, we followed the description of Dobler et al.^16^ One additional point dose value (PD^stack^) was recorded in a similar stack of RW3 phantom slabs as the 2D‐array on the axis of gantry rotation in a distance of 31 cm toward the linac structure. To investigate more points, we added the upper part of a male Alderson phantom (RSD Inc., Long Beach, CA, USA) (Fig. 1). Two slices of the phantom were substituted by similar shaped parts of PA material with bores for ionization chambers in a distance of 50.5 cm and 65.5 cm, respectively. The dose to these points is representative for the lower esophagus (PD^esoph^) and the thyroid gland (PD^thyr^). Georg et al.^10^ stated that the PD cannot easily be calculated with the TPS and Covington et al.^38^ explicitly recommend measurements. Therefore, no calculations in these points were performed. The applied ionization chambers were of type 30016 and 23332 (0.3 cm³ both), connected to Unidos dosimeters (PTW, Freiburg, Germany).

To assess the delivery times (DT), they were measured from pressing the start button to the last beam off.

## 3 RESULTS

**Figure 2:**
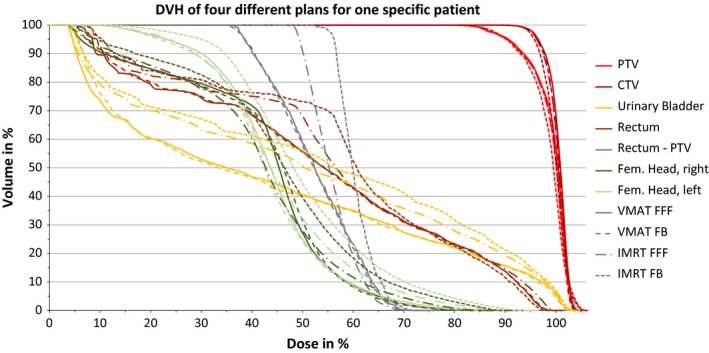
DVH for the different plans for one specific patient.

All plans met the acceptance criteria as formulated for dose prescription. Due to the fact that the focus is on the comparison of FB and FFF mode, the statistical results are grouped in Table 2 correspondingly. Differences between IMRT and VMAT are given in the text only to gain clarity. A DVH for a specific patient is shown in Fig. 2.

**Table 2:** Overview of dose volume statistics, delivery times and MU, showing average values and standard deviation

Mode		IMRT‐FB	IMRT‐FFF	VMAT‐FB	VMAT‐FFF
CTV	HI in %	8.6 ± 2.9	**8.3 ± 1.4**	7.3 ± 2.0	7.5 ± 2.0
	CI^CTV^	0.77 ± 0.06	0.81 ± 0.03	0.83 ± 0.04	**0.86 ± 0.03**
	D_av_^CTV^ in Gy	72.2 ± 0.2	72.4 ± 0.3	72.4 ± 0.3	**72.8 ± 0.1**
PTV	CI^PTV^	0.64 ± 0.07	**0.76 ± 0.09**	**0.85 ± 0.06**	0.84** ± **0.06
	D_98%_^PTV–CTV^in Gy	58.9 ± 1.4	59.1 ± 1.1	58.2 ± 1.3	**58.7 ± 1.4**
OAR	D_50%_^UB^ in Gy	41.1 ± 12.1	**37.1 ± 11.4**	32.4 ± 11.0	32.4** ± **11.2
	D_50%_^lFH^ in Gy	25.7 ± 5.0	23.4 ± 4.4	24.2 ± 5.4	23.7 ± 4.8
	D_50%_^rFH^ in Gy	25.8 ± 4.7	23.7 ± 4.9	25.7 ± 4.6	25.4 ± 4.6
	D_50%_^R^ in Gy	44.1 ± 1.7	**41.6 ± 2.6**	38.7 ± 3.5	39.1 ± 3.7
	D_2%_^Rpost^ in Gy	50.0 ± 1.1	**48.9 ± 0.8**	48.0 ± 1.1	48.4 ± 0.8
	PD^stack^ in mGy	3.6 ± 0.4	**3.0 ± 0.3**	3.4 ± 0.4	**2.5 ± 0.4**
	PD^esoph^ in mGy	1.5 ± 0.1	**1.3 ± 0.1**	1.1 ± 0.1	**0.7 ± 0.1**
	PD^thyr^ in mGy	1.3 ± 0.1	**1.1 ± 0.2**	1.2 ± 0.1	**0.6 ± 0.1**
	V_70Gy_^R^ in %	**1.8 ± 2.7**	2.7 ± 3.2	3.0 ± 2.3	**2.7 ± 3.2**
	V_60Gy_^R^ in %	**14.1 ± 6.8**	15.6 ± 6.8	15.9 ± 6.0	16.0 ± 6.2
	V_50Gy_^R^ in %	29.7 ± 6.7	28.5 ± 7.5	**27.2 ± 7.0**	27.7 ± 6.7
	V_70Gy_^UB^ in %	7.3 ± 4.4	7.1 ± 4.4	**7.3 ± 4.3**	8.1 ± 4.5
	V_65Gy_^UB^ in %	15.1 ± 6.5	13.4 ± 6.2	12.6 ± 5.9	13.3 ± 5.9
Efficiency	MU	**439 ± 23**	513 ± 42	**515 ± 57**	566 ± 33
	DT in s	294 ± 21	**276 ± 29**	84 ± 2	**77 ± 3**

The average values and sample standard deviation are over all ten plans per group. Values which are superior with statistical significance for the comparison of FB and FFF are highlighted with bold letters.

### 3.A Plan quality

The indexes for homogeneity and conformity (HI, CI^CTV^, and CI^PTV^) are either advantageous or not significantly different for FFF compared to FB with one exception: FB VMAT plans have a slightly better CI^PTV^. VMAT plans are significantly superior compared to IMRT plans.

The average dose to the CTV is very similar for all groups. There is no significance comparing VMAT and IMRT in FB mode (*P* = 0.185), but it is significantly higher for VMAT in FFF mode (*P* = 0.007).

For D_98%_^PTV–CTV^, all values are similar. Nevertheless, VMAT‐FFF was significantly higher than FB. There was no significant difference for VMAT compared to IMRT.

OAR dose values are lower for IMRT‐FFF than FB. VMAT plans are equivalent for both modes except the measured PD, which is significantly lower with FFF in the three measured points. VMAT values are either significantly lower or not significantly different compared to IMRT in the regarded OAR and PD points.

The dose values to the femoral heads (D_50%_^lFH^ and D_50%_^rFH^) are rather close and stayed at about half of the prescribed values for the objective function. Therefore, they were not further regarded according to Kjaer‐Kristoffersen et al.^39^ The volume data evaluated according the QUANTEC review^33^ show as a general result that in no single plan the given maximum percentage volume values were reached which means V_70 Gy_^R^ < 20%, V_60 Gy_^R^ < 35%, V_50 Gy_^R^ < 50%, V_70 Gy_^UB^ < 35%, and V_65 Gy_^UB^ < 35%. The average values stay far below as can be seen from Table 2. Although some differences between FB and FFF are statistically significant, they are smaller than 2% of the organ volume for the average values.

### 3.B Plan verification and efficiency

All 40 plans passed the gamma evaluation. The average passing rates in the four groups were between 98.2% and 99.5% without significant difference between FB and FFF or IMRT and VMAT.

FFF plans are delivered significantly faster than FB plans. VMAT takes less than a third of the delivery time of IMRT plans. FFF plans require more MU than FB plans and VMAT requires more than IMRT (FB and FFF *P* = 0.007 both).

## 4 DISCUSSION

The comparison of FB and FFF mode shows a clear advantage for FFF as can be seen in Table 2. Only in a few rows, there are superior values for FB. However, less MU for FB are only a technical parameter, which does not have any impact for the plan quality or patient comfort. The second advantageous parameter is the CI^PTV^ at VMAT plans. Nevertheless, the difference is very small. On the other hand, the CI^CTV^ is distinctly better for FFF mode. Third, some OAR volumes receiving a specified dose are smaller for FB than for FFF. Partly this can be traced to a smaller part of the PTV receiving the minimum dose required by the objectives and results therefore in a smaller CI^PTV^. However, these differences are small too, in most cases less than 1% of the volume of the OAR, especially in comparison to the large standard deviation. Therefore, the toxicity will be similar.

We explained in section 2.D that the minimum and maximum dose values for the CTV were set stronger than could be achieved. Therefore, the HI as documented in Table 2 does not match these limits. However, ICRU report 83^22^ states that even the limits of the ICRU report 62^40^ might be too confining for IMRT plans. Applying these limits, an even larger HI might be acceptable. There is a small advantage for FFF in IMRT (statistically significant) and a similar difference without significance for FB in VMAT. The statistically significant superiority of VMAT over IMRT is the more considerable finding regarding the HI. The prescribed minimum dose to the PTV of 59.4 Gy has not been reached by the average values of D_98%_^PTV–CTV^. As the PTV is adjacent to the rectum, the objectives for this OAR worked limiting. The maximum dose to the posterior rectum had been set to 50.0 Gy and the average values of D_2%_^Rpost^ came close up. Therefore, it must be expected that a change in the weights of these objectives might improve the minimum dose to the PTV, but increase the dose to the posterior rectum. Crijns et al.^41^ observed in their planning study for prostate treatment, that none of the RapidArc^®^ plans (with flattened beams) was within the rectum constraint.

Although the dose to the femoral heads might be improved by setting lower objectives, this could be realized only by increasing the dose from the other directions. A raised dose to the rectum or bladder would be the consequence. To avoid this, the objectives for the femoral heads were not reduced.

The plan quality of IMRT plans might increase with additional fields. Bell et al. report a standard of 11 beams.^13^ However, they did not find clinically relevant differences compared to their mArc rotation technique (which is described different from VMAT), which also resulted in shorter delivery times and therefore was recommended.

Kragl et al.^9^ and Georg et al.^10^ pointed out, that the PD is a complex function which depends on multiple factors as e.g., the distance from the primary beam. Removing the flattening filter eliminates the main source of photon scatter in the treatment head and thus explains lower measured values for FFF as it has also been reported by Dobler et al.^16^ with a similar experimental setup, Bell et al.^13^ and Dzierma et al.^11^ who measured at three positions at an Alderson phantom, and Kragl et al.^9^ performing IMRT measurements with different dosimetry systems. Similar to Dzierma et al., we found that the average values of the PD decreased with increasing distance. However, this was not valid in each single case, comparing PD^esoph^ and PD^thyr^. These dose values are especially influenced by the treatment head leakage^10^ and depend on the current collimator configuration. Closer to the isocenter, the patient scatter becomes more and more dominating.

VMAT compared to IMRT results in a further reduction of the PD which seems contradictory to the increased number of MU. This is again in accordance with the results of Dobler et al.^16^ at another localization and Bell et al.^13^ with another rotation technique (mArc). Reduced PD will result in a reduced secondary cancer risk.

As mentioned in the first paragraph of this section, the number of MU is higher for FFF compared to FB. Although it seems rather obvious that additional MU are necessary to gain a uniform dose distribution with an inhomogeneous profile, there exist contrary findings for IMRT treatment of prostate cancer with different equipment^42^ which might result from a constructional flaw in the IMRT segmentation process of the other TPS. Furthermore, it has been shown that VMAT needs more MU than IMRT. Earlier investigations using similar equipment (TPS and linac manufacturer)^18^, ^43^ confirm this result for prostate carcinoma treatment. Other studies found reverse results,^39^, ^44^, ^45^, ^46^ but referred to RapidArc^®^ compared to sliding window IMRT, which needs more MU than our step‐and‐shoot technique. For the re‐irradiation of spinal column metastasis with the same equipment as the present study,^16^ there was no difference in the number of MU between IMRT and VMAT which can be taken as an example that different targets must be investigated individually.

It has been mentioned in the introduction that FFF aimed to shorter treatment times especially for radiosurgical applications. The benefit which is demonstrated in our investigation is rather small compared to the enabled maximum dose rate. The first reason is, that more MU are required per fraction dose for FFF than for FB as described above. For VMAT, additionally higher dose rate values than possible with FB are not exploited between all control points of the whole arc as there are limiting factors like leaf speed and gantry speed. Thus, the delivery time of VMAT‐FFF is 8% shorter than for FB and yields a further reduction of the probability for intrafraction movement of the target. VMAT in both modes is therefore well in the time interval of 2–3 min where no additional position verification and correction is required.^47^

## 5 CONCLUSIONS

Taking all results into account, for the given equipment and the treatment of patients with prostate carcinoma, a VMAT technique with FFF mode is recommended. It combines the best plan quality with fastest delivery and lowest PD.

## CONFLICT OF INTEREST

The department had a research cooperation with Elekta GmbH, Hamburg, Germany which ended in March 2017.
